# Comparative analysis of cranberry fruit rot fungal diversity in Massachusetts from wild, organic, and conventional ecosystems using multiplex PCR

**DOI:** 10.3389/fpls.2025.1500877

**Published:** 2025-08-15

**Authors:** Salisu Sulley, Mura Jyostna Devi, Benjamin Cinget, Matteo Conti, Richard Bélanger, Marty Sylvia, Frank Caruso, Leela Saisree Uppala

**Affiliations:** ^1^ Cranberry Station, University of Massachusetts-Amherst, East Wareham, MA, United States; ^2^ Vegetable Crops Research Unit, United States Department Agriculture-Agriculture Research Services, Department of Plant and Agroecosystem Sciences, University of Wisconsin-Madison, Madison, WI, United States; ^3^ Department of Plant Science, Université Laval, Québec, QC, Canada

**Keywords:** cranberry fruit rot (CFR), fungal diversity, multiplex PCR, ecosystems, species richness

## Abstract

Cranberry fruit rot (CFR) has been a major challenge in cranberry production affecting fruit quality, particularly in Massachusetts and New Jersey. It is known to be a disease complex associated with several diverse fungi. This study provides the first comprehensive assessment of CFR fungal population dynamics across wild, organic, and conventional cranberry production systems in southeastern Massachusetts. By employing multiplex PCR, a high-throughput molecular method for the simultaneous detection of multiple fungi, we investigated the prevalence and diversity of 11 most commonly associated fruit rot fungi in 2021 and 2022, in 32 (23 conventional, 4 organic and 5 wild) and 50 (40 conventional, 4 organic and 6 wild) cranberry bogs respectively. Significant differences in the detection of CFR fungi were observed across these ecosystems. Conventional fields showed varied fruit rot incidence, ranging from 2-42% in 2021 and 1-48% in 2022. Species richness analysis indicated that on average, wild bogs were more species-rich and diverse, with 5.5 CFR fungi detected per sample compared to 4 CFR fungi in both conventional and organic bogs. Organic bogs exhibited a significant decline in species richness from 6 fungi in 2021 to 2 fungi in 2022. Except for *Phomopsis vaccinii*, which was not observed in organic bogs in either year, all 11 CFR fungi were detected across the ecosystems: some in both growing seasons and some in only one. Key CFR fungi such as *Coleophoma empetri*, *Allantophomopsis cytisporea and Botryosphaeria vaccinii* were consistently found across all ecosystems and both years of study. Analysis of year-to-year variation indicates that wild bogs exhibited higher fungal carryover, with 8 out of the 11 CFR fungi persisting from one year to the next, compared to 6 fungi in conventional bogs and 4 fungi in organic bogs. Notably, *C. empetri*, *B. vaccinii*, and *A. cytisporea* demonstrated year-to-year persistence across all three bog types. Among these, *C. empetri* exhibited the greatest persistence across all three ecosystems, with 96%, 100% and 60% of the conventional, organic and wild bogs maintaining its presence across both years. *Colletotrichum* spp. persisted in 43% of the conventional bogs but detected only in one of the two years studied in organic and wild bogs resulting in 0% persistence from one year to the next. The study also noted a general decrease in *Botryosphaeria vaccinii* and an increase in *Physalospora vaccinii* across all bog types. This research highlights the varying persistence and complex dynamics of CFR fungi across different cranberry ecosystems, as well as the potential impact of management practices on fungal populations.

## 1 Introduction

The domestication of the American cranberry (*Vaccinium macrocarpon* Ait.) in the United States traces its roots back to the early nineteenth century, as documented by Eck (1990); Eastwood (1956), and White (1885). As of 2022, global cranberry production totaled approximately 582,924 tonnes, with the United States producing about 365,500 t (~62.7 %), and Canada producing 209,205 t (~35.9 %) (Wikipedia, 2025; World Population Review, 2025). Cranberry production in the US is concentrated in five states (Massachusetts, New Jersey, Oregon, Washington and Wisconsin) with Wisconsin being the lead producer, followed by Massachusetts (USDA-NASS, 2022). Cranberries maintain their status as Massachusetts’ most economically valuable crop, and the majority of cranberry bogs, as the cranberry fields are commonly referred to in Massachusetts, are concentrated in the southeastern part of the state (Averill et al., 2008).

The growing interest in cranberries can be attributed to the increasing awareness of their perceived health benefits (Andersen, 1989; Brown et al., 2012; Hisano et al., 2012; Santillo and Lowe, 2007; Mantzorou and Giaginis, 2018). The availability of various processed products, such as sweet and dried cranberries, as well as juices, further adds to the appeal of this fruit (USDA-FAS, 2016). The economic impact of cranberry cultivation—including production, processing, and industry support services—is estimated at $1.7 billion (Farm Credit East, 2023). In recognition of this significance, Massachusetts Governor Maura Healey and Lieutenant Governor Kim Driscoll’s Administration officially designated October as “Massachusetts Cranberry Month” to honor the crop’s contribution to the state’s economy (MDAR, 2023).

Since the turn of the millennium, cranberry cultivation across all growing regions has encountered declining profitability, despite a rising demand. Several factors contribute to this trend, including historically low prices, increasing production costs, stringent fruit quality standards, and concerns related to both biotic and abiotic stresses (Averill et al., 2008; Hira et al., 2003; Hoekstra et al., 2020). Diseases, particularly cranberry fruit rot (CFR), a term used to describe general softening and deterioration of cranberries in the field and storage, pose a significant obstacle to cranberry production, resulting in substantial economic losses for growers, especially in Massachusetts and New Jersey (Caruso et al., 2000; Oudemans et al., 1998; Polashock et al., 2009). These two northeastern states face heightened fruit rot pressure compared to other growing regions, in part due to elevated summer temperatures (Eck, 1990; Oudemans et al., 1998; Tadych et al., 2015; Wood et al., 2023). Research on cranberry fruit rot began in the early 1900s, revealing the involvement of several taxonomically diverse fungal species, making it a complex disease (Bain, 1926; Caruso et al., 2000; Conti et al., 2022; McManus et al., 2003; Olatinwo et al., 2003; Stevens, 1924, 1932; Vilka and Bankina, 2013; Zuckerman, 1958).

The distribution of CFR fungal species, along with the incidence and severity of resulting fruit rot, is documented to vary among cultivars, geographical locations, growing seasons, and phenological stages (Bergman and Wilcox, 1936; McManus et al., 2003; Oudemans et al., 1998; Polashock et al., 2009; Stevens, 1924; Wells-Hansen and McManus, 2017; Zuckerman, 1958). Based on the literature, 10–15 species, including *Colletotrichum fioriniae* (Marcelino & Gouli ex R.G. Shivas & Y.P. Tan; teleomorph *Glomerella acutata* var. *fiorinae*), *Colletotrichum fructivorum* (V.P. Doyle, P.V. Oudem. & S.A. Rehner; teleomorph *Glomerella cingulata*), *Coleophoma empetri* Rostr. (syn *C. cylindrospora*), *Phyllosticta vaccinii* (Earle*), Physalospora vaccinii* Shear (Arx & E. Müll), *Allantophomopsis lycopodina* (Höhn) Carris, *Allantophomopsis cytisporea* (Fr.) Petr., *Strasseria geniculata* (Berk. & Broome) Höhn, *Botryosphaeria vaccinii* (anamorph *Phyllosticta elongata* Weid.), *Godronia cassandrae* (anamorph *Fusicoccum putrefaciens* Shear), *Monilinia oxycocci* (Woronin) Honey, and *Diaporthe vaccinii* (anamorph *Phomopsis vaccinii* Shear, are known to be the most prevalent CFR fungi across growing regions (Damm et al., 2012; McManus et al., 2003; Oudemans et al., 1998; Polashock et al., 2009; Weir et al., 2012). Each of these species is associated with a specific fruit rot disease (bitter rot, ripe rot, early rot, blotch rot, black rot, berry speckle, end rot, cotton ball, viscid rot) that make up the CFR disease complex.

An accurate understanding of the fungi associated with cranberry fruit rot (CFR) and their role in disease development is essential for the strategic management of CFR. Currently, comprehensive data on the distribution and diversity of the CFR complex across organic, conventional, and wild cranberry ecosystems in Massachusetts is lacking. We hypothesize that gradual climate shifts, the movement of plant materials, and the introduction of high-yielding cranberry hybrids may have facilitated the introduction, establishment, spread, and redistribution of these fungi over time. Furthermore, as artificial selection through plant domestication is known to alter various aspects of the plant microbiome, it is reasonable to question how CFR-associated fungal communities differ in wild cranberry bogs—which, anecdotally, produce smaller berries with lower levels of fruit rot. Therefore, gaining a thorough understanding of the causal agents contributing to fruit rot in wild, organic, and conventional cranberry systems is imperative.

Traditionally, characterization of CFR fungi has been based on plating of symptomatic rotten fruits, isolation of fungal pure cultures and microscopic identification based on morphological characteristics (Bergman and Wilcox, 1936; Jeffers, 1991; Stevens, 1924; Stretch and Ceponis, 1986; Zuckerman, 1958). Due to the coexistence of multiple fungi in each sample, during plating, the fast-growing fungi may suppress the growth of slow growing fungi. Additionally, morphological characterization may result in inaccuracies particularly among the closely related species. Over the past two decades, the adoption of molecular tools such as polymerase chain reaction (PCR) and sequencing for fungal identification using primers targeting internal transcribed spacer (ITS) regions (Polashock et al., 2017; Stiles and Oudemans, 1999; Wells-Hansen and McManus, 2017) has significantly improved accuracy. The development of multiplex PCR (Conti et al., 2019) has streamlined the rapid and simultaneous identification of all prevalent CFR fungi, saving both time and resources (Conti et al., 2019, 2020, 2022).

The current study was initiated with an objective to understand the current status of the principal fungi associated with CFR from organic, wild and conventional Massachusetts bogs.

## 2 Materials and methods

### 2.1 Field sites and sampling details

**Figure 1:**
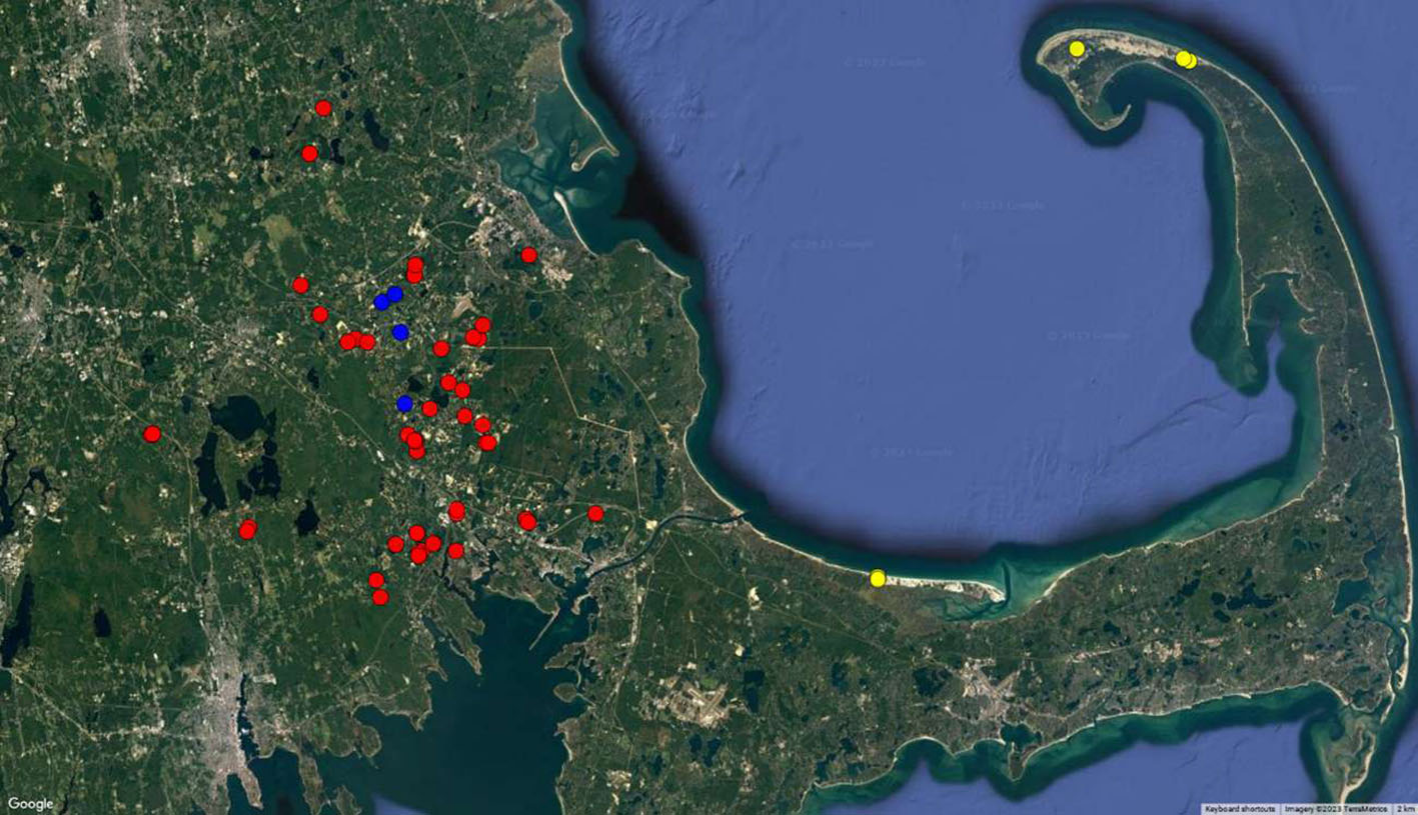
Distribution of cranberry bogs sampled for this study. The surveyed bogs are located in southeastern Massachusetts. Cranberry bogs are color-coded according to the eco systems. Red, Blue, and Yellow indicate the locations of conventional, organic, and wild bogs sampled, respectively. Conventional and organic bogs are located in Plymouth County while wild bogs are located in Barnstable County.

In 2021 and 2022 growing seasons, 32 (23 conventional, 4 organic and 5 wild) and 50 (40 conventional, 4 organic and 6 wild) cranberry bogs located in southeastern Massachusetts, were surveyed, and sampled for symptomatic rotten cranberries, respectively. In this manuscript, the conventional, organic, and wild bogs are referred to by the abbreviations C, O, and W, respectively. Samples were collected from conventional bogs (that were sprayed with registered fungicides for fruit rot) cultivated with “Stevens” cultivar, spread across two counties and eight cities in this region: Plymouth County (Carver, Halifax, Hanson, Lakeville, Middleborough, Rochester, and Wareham) and Bristol County (Freetown) (**Figure 1**).

In both years of the study, berries were sampled at “harvest maturity” in late September or the first week of October, just prior to commercial harvest. From each bog, five replicate samples were collected. Each replicate consisted of all berries within a randomly selected 0.3 m² (1-square-foot) quadrant, excluding areas along the bog edges. Berries were handpicked, placed in paper bags, and transported to the laboratory for further processing, which included evaluation of CFR incidence and multiplex fungal characterization (detailed in subsequent sections).

The current study also included four organic bogs cultivated with the ‘Early Blacks’ cultivar, located in Carver, MA; four wild cranberry bogs situated in the Province Lands within the Cape Cod National Seashore (CCNS)—along trails spanning the hilly sand dunes, Herring Cove Beach, and Race Point Beach; and two additional wild bogs located near Sandy Neck Park. Rotten berries from the organic bogs were obtained during the sorting and processing stage from a collaborating grower after the bogs had already been harvested, while those from the wild bogs were collected randomly during a walk through the bogs in late September to early October.

### 2.2 Evaluation of CFR incidence from collected samples

To determine the percent rot incidence from conventional bogs in our study, the berries brought to the lab were first passed through a sieve with 5.6 mm opening/mesh size to separate the pinheads (that pass through the sieve) from the industry standard sized (that are retained on top of the sieve) berries. Subsequently, the industry standard sized berries were visually examined to separate asymptomatic visually healthy and symptomatic (rotten) berries. Rotten berries were counted to determine the fruit rot incidence (% Rot) by using the following formula:


$$\%\;Rot\;Incidence=\frac{Number\;of\;Rotten\;Berries}{Total\;Number\;of\;Berries\;}\times 100$$

Following this, rotten berries from all five replicates of a specific bog were combined to create one composite sample. These composite samples were then stored at -20°C until DNA isolation.

However, % Rot incidence was not assessed from the samples collected from organic and wild fields. This was either because organic cranberry fields were harvested earlier, or we lacked access to them prior to harvest. In the case of wild fields, their unstructured nature- characterized by irregular plant density, uneven ground surfaces and inconsistent patterns of fruit distribution made it impractical/challenging to implement standardized sampling methods to conduct a reasonable or accurate assessment of fruit rot incidence.

### 2.3 CFR fungal DNA isolation

To ensure the PCR detection to the CFR species only, the total genomic DNA of the rotten berries was isolated following the protocol outlined by Conti et al. (2019). Twenty rotten berries were randomly selected from the composite rotten berries of each bog and split into five replications (i.e., four berries per replication). The berries were surface sterilized (5 min in 0.5% NaOCl and rinsed three times in sterile distilled water) and lyophilized. The lyophilized berries were then ground using sterilized beads, 100 mg of each ground sample was weighed and utilized for DNA extraction.

For this, 500 ml of CTAB extraction buffer was added to the 100 mg sample and vortexed. An additional 700 ml of the CTAB buffer was added, and the mixture was incubated in a 65°C water bath for 10 minutes, followed by centrifugation at 10,000 g for 10 minutes. From the resultant supernatant, 700 ml of supernatant were transferred and mixed with 525 ml of guanidine hydrochloride. The mixture was centrifuged in a column spin and the DNA was washed twice in 70% ethanol. Finally, the DNA was eluted with 100 ml of elution buffer. The purity and concentration of the DNA was measured using a Nanodrop One C spectrophotometer (Thermo Scientific, Madison, WI) and then stored at -20°C.

### 2.4 Multiplex PCR for identifying the most prevalent CFR fungi

A multiplex PCR (mPCR), consisting of four reactions, each targeting a specific set of CFR fungi, was performed following the protocol proposed by Conti et al. (2019), with modifications in some primer sequences to improve the accuracy of detection in the context of this study. All modified primers were experimentally validated for specificity and efficacy using genomic DNA from morphologically and molecularly confirmed CFR isolates. These isolates were sourced from both field-collected cranberry samples in Massachusetts and Quebec and from recognized fungal culture collections, including the American Type Culture Collection (ATCC), the Canadian Collection of Fungal Cultures (DAOM), Central Bureau of Fungal Cultures-Royal Netherlands Academy of Arts and Sciences (CBS-KNAW), and the Philip E. Marucci Center. Primer sets were tested for amplification of the correct fragment lengths under the cycling conditions outlined in **Table 1**, with representative amplicons sequenced via Sanger sequencing to confirm identity. To assess potential cross-reactivity, primers were also tested against non-target CFR species listed in **Supplementary Table 1**, and no off-target amplification was observed. The primer list, reaction mixes, target fungi, and expected amplicon sizes are detailed in **Table 2**. Each reaction was performed in a total volume of 25 μl per sample, consisting of 12.75 μl Milli-Q water, 5 μl PCR buffer, 0.5 μl of 10 mM dNTP, 2 μl of primer mix, 0.625 μl DMSO, 0.125 μl OneTaq 5U/μl, and 4 μl of DNA template.

**Table 1:** PCR cycling conditions.

Reaction	Step	Time	T°C	Cycles
Mix A	Initial denaturation	5 minutes	94	
	Denaturation	30 sec	94	40
	Annealing	30 sec	65	
	Elongation	1 minute	68	
	Final elongation	5 minutes	68	
	Rest	∞	12	
Mix B	Initial denaturation	5 minutes	94	
	Denaturation	30 sec	94	40
	Annealing	30 sec	62	
	Elongation	45 sec	68	
	Final elongation	5 minutes	68	
	Rest	∞	12	
Mix C	Initial denaturation	5 minutes	94	
	Denaturation	30 sec	94	40
	Annealing	30 sec	51	
	Elongation	30 sec	68	
	Final elongation	5 minutes	68	
	Rest	∞	12	
Mix G	Initial denaturation	5 minutes	94	
	Denaturation	30 sec	94	40
	Annealing	30 sec	51	
	Elongation	40 sec	68	
	Final elongation	5 minutes	68	
	Rest	∞	12	

**Table 2:** The multiplex reaction details, target fungi, primers (forward and reverse) and expected amplicon sizes.

Reaction	Target fungi	Primer name	Sequence	Amplicon	Reference
Mix A	Phacidiaceae	Phacidiaceae_1_fw	GTCGCAAGACAACCGGC	396-414	Conti et al., 2019
		fruitrot_rv	CCTACCTGATCCGAGGTCAAC		Conti et al., 2019
	*Monilinia oxycocci*	SP8_fw_d	CCACAGGGGCAGAACCTCTC	458	Conti et al., 2019
		fruitrot_rv	CCTACCTGATCCGAGGTCAAC		Conti et al., 2019
	*Phyllosticta vaccinii*	P vaccinii_fw	CAGCACCCCTTGTGTACC	518	Conti et al., 2019
		Fruitrot_rv	CCTACCTGATCCGAGGTCAAC		Conti et al., 2019
Mix B	*Botryosphaeria vaccinii*	MixB_Fw	CCTACCTGATCCGAGGTCA	590	This study
		Botryosphaeria_rv	AAAGCTCCCCTGGTACATGC		This study
	*Coleophoma empetri*	MixB_Fw	CCTACCTGATCCGAGGTCA	105	This study
		Coleophoma_rv	CCGTCTGGCTCTAAGCGTAG		This study
	*Colletotrichum spp.*	MixB_Fw	CCTACCTGATCCGAGGTCA	380	This study
		Colletotrichum_rv	ACGTCTCTTCTGAGTGGCAC		This study
	*Phomopsis spp.*	MixB_Fw	CCTACCTGATCCGAGGTCA	145	This study
		Phomopsis_rv	AAGGCAGGCCCTGAAATTCA		This study
	*Physalospora vaccinii*	MixB_Fw	CCTACCTGATCCGAGGTCA	480	This study
		Physalospora_rv	CGCTCAAAAACAACGCGAAC		This study
Mix C	*Allantophomopsis cytisporea*	SP1_BR_fw	CTCAGCGGCTTCCTGAATA	195	Conti et al., 2019
		SP1_BR_rv	CGTCTTTTGCCCCTCTACTC		This study
	*A. lycopodina*	SP2_BR_fw	ATAATTCATGGCCCGAGGTA	150	This study
		SP2_BR_rv	TCTTTTTGCCCCTCCTACAG		This study
	*Stressaria geniculata*	SP12_BR_fw	GCGGCTTCCTGTTTATTGAA	170	This study
		SP12_BR_rv	GCCCCTCTACTACGCTTGTG		This study
Mix G	Universal Cranberry	vm_Cytb fwd	GATACGTACTACCTTGGG	380	This study
		vm_Cytb rev	AATATGAGGCGGGGTGGA		This study
	*Godronia cassandrae*	Godronia_Fw	ATCTCCCAGCTCTTAAAATC	363	This study
		Godronia_rv	TCACATGAGCGAAAGCAC		This study

The cycling conditions for PCR mixes are presented in **Table 1**. The PCR products were analyzed using the QIAxcel Advanced System (Qiagen, Hilden, Germany) with the QIAxcel DNA High-Resolution Kit and the preinstalled OM500 method, which includes 15 bps and 3000 bps alignment markers.

Proportion of CFR fungal prevalence: For each CFR fungi under multiplex consideration, % fungal prevalence was determined by the formula:


$$\begin{array}{c} \%\;Fungal\;Prevalence=\\ \frac{Number\;of\;fields\;Infected\;with\;a\;particular\;CFR\;pathogen}{Total\;Number\;of\;Fields\;Sampled}\times 100 \end{array}$$

Species relative abundance (SRA) was calculated as the proportion of each CFR species in relation to the total number of CFR species detections for each bog type. It was estimated from mPCR results with the following formula:


$$\begin{array}{c} \;Species\;Relative\;Abundance\;=\\ \frac{Sum\;of\;positives\;for\;a\;given\;CFR\;pathogen}{Sum\;of\;all\;positive\;CFR\;pathogen\;detections}\times 100 \end{array}$$

*Species Diversity*: The species diversity was estimated for each sampling field as “the number of CFR species simultaneously detected by mPCR in a given field. The species diversity scale in this study ranged from 0-11.

### 2.5 Year-to-year variations in CFR fungi

Out of the total bogs studied, 32 were sampled both years (23 conventional bogs, 4 organic bogs and 5 wild bogs). They were studied for year-to-year variations in CFR fungal abundance and incidence.

### 2.6 Data analysis

To determine the effects of the field type (conventional, organic, and wild), and growing seasons (2021 and 2022) from which samples were collected on fungal profiles, relative abundance data were analyzed using the negative binomial with inflated zero model in the glmmTMB package (Magnusson et al., 2017) in R Statistical Software version v4.2.3 (R Core Team, 2021). Model appropriateness was assessed using DHARMa package (Hartig, 2018) in R.

Negative binomial model with inflated zero


$$Y_{i}∼\text{ ZINM}\left(\lambda_{i,}\theta_{i},\pi_{i}\right)$$

Where $Y_{i}$ represents the abundance for the $i^{th}$ observation (dependent variable), $\lambda_{i}$ is the mean of the Negative Binomial distribution for observation i , $\theta_{i}$ is the dispersion parameter of the Negative Binomial distribution for observation i and $\pi_{i}$ is the probability of the excess zero outcome.

Zero-Inflation Component


$$log\left(\frac{\pi_{i}}{1-\pi_{i}}\right)=\beta_{0}+B_{1}Year_{i}+B_{2}FieldType_{i}$$

Where $\beta_{0}$ is the intercept, $\beta_{1}$ and $\beta_{2}$ are the coefficients for the fixed effects for Year and Field Type.

Negative Binomial component


$$log\left(\lambda_{i}\right)=\gamma_{0}+\gamma_{1}Year_{i}+\gamma_{2}FieldType_{i}+\mu_{Species,i}+\mu_{Bog,i}$$

Where $\gamma_{0}$ is the intercept, $\gamma_{1}$ and $\gamma_{2}$ are the coefficients for the fixed effects Year and Field Type. $\mu_{Species,i}$ and $\mu_{Bog,i}$ represent the random effects for Species and Bog for the $i^{th}$ observation, respectively. The random effects $\mu_{Species,i}$ and $\mu_{Bog,i}$ follow normal distributions with mean zero and variances $\sigma_{Species}^{2}$ and $\sigma_{Bog}^{2}$ , respectively.

Alpha Diversity Analysis

To assess within-sample fungal diversity, we calculated the Shannon diversity index using the abundance data of 11 fungi identified across conventional, organic, and wild cranberry systems (bog types). All diversity analyses were conducted in R (v4.3.0) using the vegan package (Oksanen et al., 2007). Shannon diversity values were compared among bog types using the Kruskal–Wallis rank-sum test, followed by pairwise Wilcoxon tests with Benjamini–Hochberg.

Beta Diversity Analysis

To evaluate differences in fungal community composition among conventional, organic, and wild cranberry systems (bog types), we calculated Bray–Curtis dissimilarities and assessed group-level differences using permutational multivariate analysis of variance (PERMANOVA) with 999 permutations. Principal Coordinates Analysis (PCoA) was used to visualize the compositional dissimilarity among samples. Group separation was illustrated along the first two principal coordinates, with 95% confidence ellipses overlaid to depict variability within each bog type.

Year-to-Year Comparison of Fungal Abundance through heatmaps

For year-to-year variation study, two separate heatmaps representing data for 2021 and 2022, were generated for the 32 bogs that were studied both years, in R programming. A color gradient ranging from blue to red was applied to visualize fungal abundance in individual bogs. Blue represented lower abundance, while red indicated higher abundance. This gradient was scaled based on the relative abundance values for each fungus-bog pair (0–5 scale representing how many replications were positive). Hierarchical clustering was performed on both axes (fungal species and cranberry bogs). This clustering grouped similar fungal abundance patterns across the bogs and fungal species, and dendrograms were included on both axes to show the clustering. To assess year-to-year carryover and the consistency of CFR fungal prevalence across seasons, we analyzed the proportion of bogs in which any of the 11 targeted fungi were consistently detected in both study years. The results were expressed as percentages.

## 3 Results

### 3.1 CFR fungi across cranberry systems (conventional, organic, wild):

Multiplex PCR data (**Table 3**) revealed key differences in the proportion of individual CFR fungal prevalence, across cranberry systems (conventional, organic, and wild bogs) and sampling years (2021 and 2022).

*Conventional fields*: Among the conventional cranberry fields, the % Rot incidence varied substantially across sampling locations and sampling years. In 2021, the % Rot incidence ranged from 2 to 42%, while in 2022, it ranged from 1 to 48% (**Table 4**). The prevalence of CFR fungi also varied from year to year (**Table 3**). However, in both years, *C. empetri* was detected in more than 90% of the fields, with rates of 92% and 100% in 2021 and 2022, respectively. In 2021, black rots fungi (*A. cytisporea*, *A. lycopodina* and *S. geniculata*) were detected in 50% of the conventional fields, which increased to 67% in 2022. Among the three black rot-associated fungi, *A. cytisporea* was the most prevalent, isolated from 42% of fields in 2021 and 54% in 2022. The prevalence of *S. geniculata* was approximately 8% in 2021 but rose sharply to 28% in 2022. Blotch rot fungi *Physalospora vaccinii* (*Phys. vaccinii*), went from 0% in 2021 to 72% in 2022, making it the CFR fungi with the most significant shift. Viscid rot fungi *Phomopsis vaccinii* (*Phom. vaccinii*), also rose notably from 21% in 2021 to 44% in 2022. On the contrary, *Colletotrichum* spp. associated with bitter rot, *G. cassandrae* associated with end rot, *Phyllostica vaccinii* (*Phyl. vaccinii*) associated with early rot and *M. oxycocci* associated with cottonball decreased in occurrence between 2021 and 2022. (**Table 3**).

*Organic bogs*: Similar to conventional bogs, *C. empetri* was the predominant CFR fungi in organic bogs, being detected in all bogs in both 2021 and 2022. Also, *A. cytisporea* was the predominant black rot fungi during both the study years. Interestingly, *A. lycopodina*, *S. geniculata*, *Colletotrichum* spp., *G. cassandrae*, *M. oxococci*, *Phyl. vaccinii*, were not isolated in any of the organic bogs in 2022, despite being isolated from some organic bogs in 2021. *Phom. vaccinii* on the other hand was not detected in either year. The percentage of organic fields from which *Phys. vaccinii* was isolated increased from 25% in 2021 to 50% in 2022 (**Table 3**).

*Wild fields*: *C. empetri* was detected in all wild bogs in 2021 but was only detected from 67% of bogs in 2022. The percentage of wild bogs detected with black rot associated fungi increased from 2021 (75%) and 2022 (83%). *A. lycopodina* was the predominant black rot fungus in 2021, while *A. cytisporea* was the predominant one in 2022 among wild bogs. *A. cytisporea*’s prevalence significantly increased from 25% in 2021 to 83% in 2022. *S. geniculata* and *Colletotrichum* spp. were not isolated from the wild bogs in 2021, while *G. cassandrae* and *M. oxycocci* were not isolated in 2022 (**Table 3**). Prevalence of *Phys. vaccinii* increased from 25% to 67% between 2021 and 2022.

**Table 3:** Prevalence of individual CFR fungi and associated Rot types in 2021 and 2022 among Conventional, Organic and Wild Cranberry Ecosystems in Massachusetts.

Ecosystem	Fungal species	CFR fungal Prevalence (%)				Disease/Rot types
		2021		2022		
Conventional	*Allantophomopsis cytisporea*	48	50*	54	67*	Black rot
	*Allantophomopsis lycopodina*	21		26		
	*Strasseria geniculata*	8		28		
	*Botryosphaeria vaccinii*	54		13		Berry speckle
	*Coleophoma empetri*	92		100		Ripe rot
	*Colletotrichum spp.*	75		56		Bitter rot
	*Godronia cassandrae*	17		10		End rot
	*Monilinia oxycocci*	29		10		Cottonball
	*Phomopsis vaccinii*	21		44		Viscid rot
	*Phyllosticta vaccinii*	8		5		Early rot
	*Physalospora vaccinii*	0		72		Blotch rot
Organic	*Allantophomopsis cytisporea*	75	75*	50	50*	Black rot
	*Allantophomopsis lycopodina*	50		0		
	*Strasseria geniculata*	25		0		
	*Botryosphaeria vaccinii*	50		50		Berry speckle
	*Coleophoma empetri*	100		100		Ripe rot
	*Colletotrichum spp.*	75		0		Bitter rot
	*Godronia cassandrae*	25		0		End rot
	*Monilinia oxycocci*	50		0		Cottonball
	*Phomopsis vaccinii*	0		0		Viscid rot
	*Phyllosticta vaccinii*	50		0		Early rot
	*Physalospora vaccinii*	25		50		Blotch rot
Wild	*Allantophomopsis cytisporea*	25	75*	83	83*	Black rot
	*Allantophomopsis lycopodina*	50		67		
	*Strasseria geniculata*	0		50		
	*Botryosphaeria vaccinii*	50		67		Berry speckle
	*Coleophoma empetri*	100		67		Ripe rot
	*Colletotrichum spp.*	0		33		Bitter rot
	*Godronia cassandrae*	75		0		End rot
	*Monilinia oxycocci*	25		0		Cottonball
	*Phomopsis vaccinii*	25		33		Viscid rot
	*Phyllosticta vaccinii*	75		33		Early rot
	*Physalospora vaccinii*	25		67		Blotch rot

*Indicates the average Percent prevalence for the three black rot fungi—*Allantophomopsis cytisporea*, *Allantophomopsis lycopodina*, and *Strasseria geniculata*—within a specific study year and bog type.

**Table 4:** Cranberry fruit rot incidence among the conventional bogs during 2021 and 2022 growing seasons.

Sampling city	Percent fruit rot (%)	
	2021	2022
Carver	14 – 38 (7^*^)	1 – 31 (12)
Freetown	18 (1)	1 – 16 (2)
Halifax	N/A^**^	1 – 19 (2)
Hanson	N/A	14 (1)
Lakeville	42 (1)	2 – 48 (2)
Middleborough	8 – 18 (5)	1 – 22 (8)
Rochester	2 – 4 (2)	
Wareham	8 – 20 (7)	1 – 47 (11)

*The number in parentheses represents the number of fields sampled from a particular city.

**No fruit rot incidence was measured in beds.

### 3.2 CFR species relative abundance

Using the negative binomial model with inflated zero, the SRA did not differ significantly (*P =* 0.12) across bog types. Conversely, variation in the SRA over the study period (between 2021 and 2022) (*P* = 0.04) and bog type by year interaction were significant (*P* = 0.008) (**Supplementary Table 2**). Specifically, the interaction effect of year and bog was significant (*P* = 0.002) among organic bogs.

Over the two years of the study, the four most detected species in terms of relative species abundance were *C. empetri*, *Colletotrichum* spp., *B. vaccinii* and *Phys. vaccinii*. Consistently, *C. empetri* was the most abundant in both years. However, while *Colletotrichum* spp. was second to *C. empetri* in 2021, *Phys. vaccinii* was second to *C. empetri.* in 2022. Furthermore, when considered collectively, the fungi associated with black rot (*Allantophomopsis cytisporea*, *A. lycopodina*, and *Strasseria geniculata*) represented the second most abundant group.

**Figure 2:**
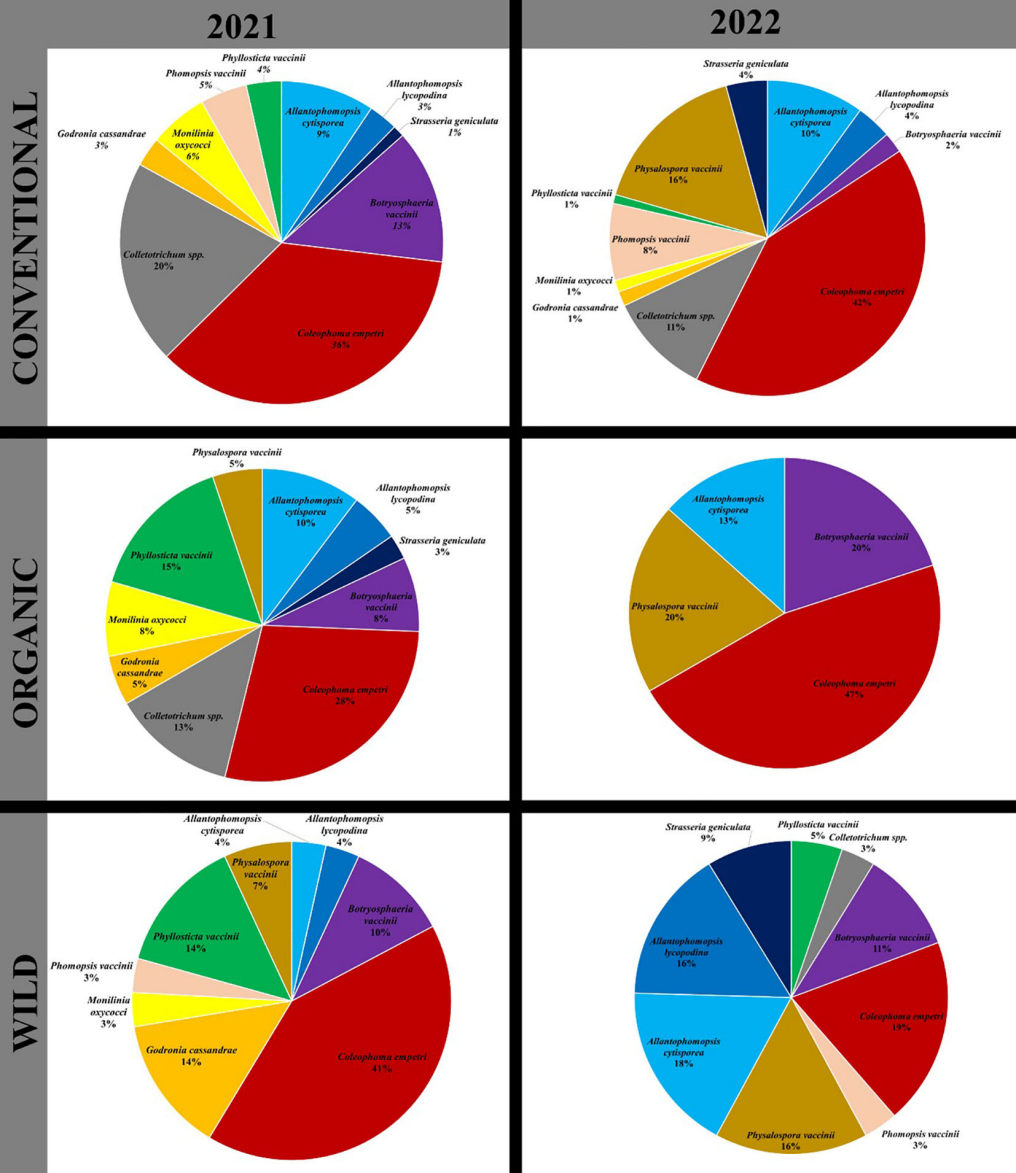
Species relative abundance among Conventional, Organic and Wild cranberry systems for 2021 and 2022 growing seasons. Pie chart represents the relative abundance of a CFR fungal species in a bog type in a growing season.

In both 2021 and 2022, *C. empetri* was the predominant CFR fungus detected in cranberry bogs in Massachusetts, representing SRA of 36% and 42%, respectively, in the conventional bogs; 41% and 19%, respectively, in the wild bogs; and 28% and 47% in the organic bogs (**Figure 2**). The relative abundance of *Physalospora vaccinii* increased in 2022 across all bog types compared to 2021. In conventional, organic, and wild bogs, its species relative abundance (SRA) rose from 0%, 5%, and 7% in 2021 to 16%, 20%, and 16% in 2022, respectively. Conversely, the SRA of *Phyl. vaccinii* decreased in 2022, from 4% to 1%; 15% to 0%; and 14% to 5%, for conventional, organic, and wild bogs, respectively.

### 3.3 CFR species richness

At least one of the CFR species was isolated in more than 90% of the fields sampled. Notably, a maximum of nine fungi were simultaneously detected in a single sample from the wild bog in 2022. In conventional fields, the average number of CFR fungi isolated in a sample remained consistent at “4” for both 2021 and 2022. However, in organic fields, there was a notable difference in the average number of fungi detected, with a significant reduction from 6 fungi in 2021 to 2 fungi in 2022 (**Supplementary Figure 3**). In wild bogs, the average number of CFR fungi isolated remained similar at 5.5 in 2021 and 2022. However, the species richness exhibited a wide variation in 2022 compared to 2021 (**Supplementary Figure 3**).

**Figure 3:**
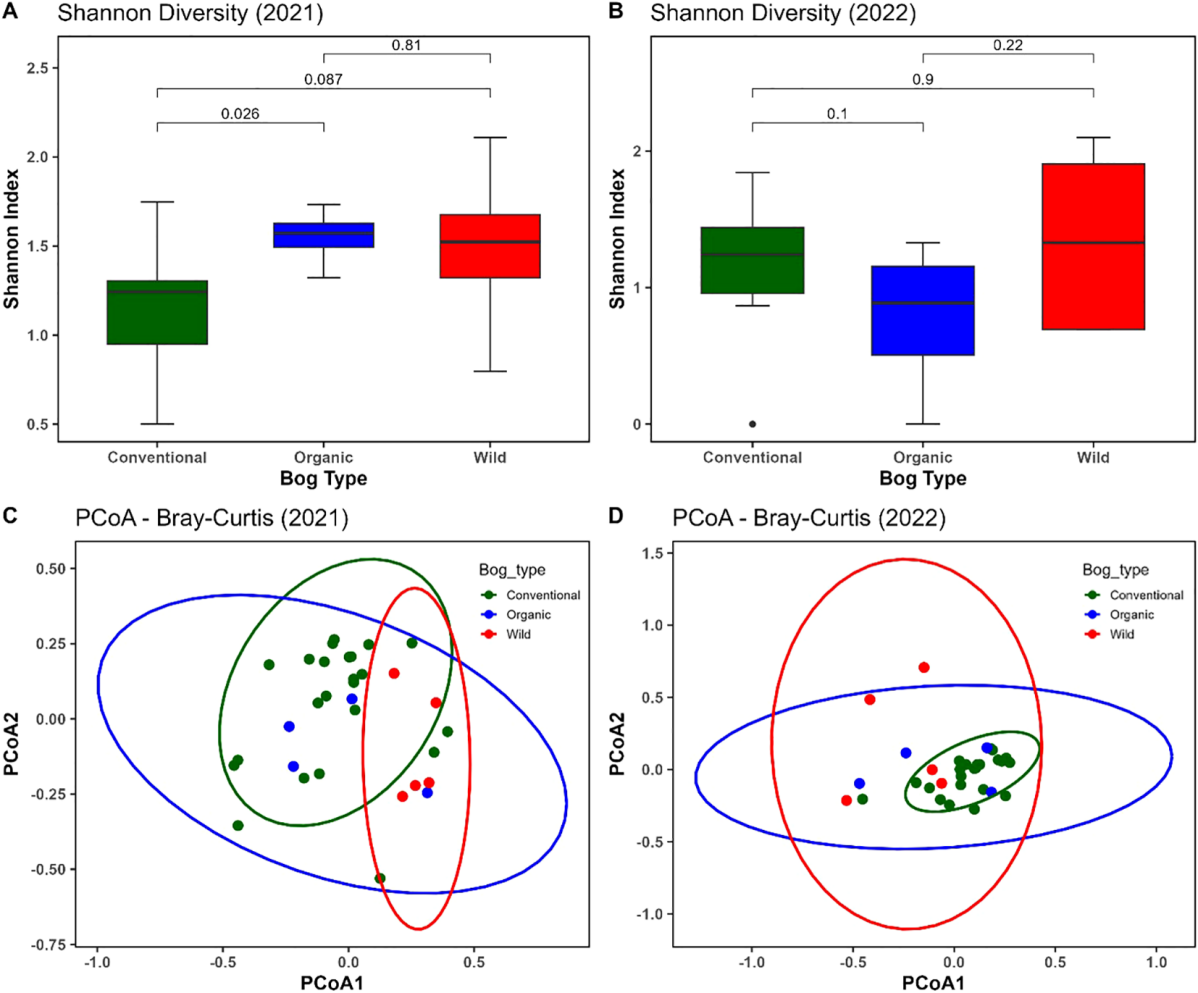
Alpha and beta diversity of fungal communities across bog types in 2021 and 2022. **(A, B)** Shannon diversity indices across conventional (green), organic (blue), and wild (red) cranberry bogs in 2021 **(A)** and 2022 **(B)**. Significant differences between groups were assessed using the Kruskal–Wallis test followed by pairwise comparisons. **(C, D)** Principal Coordinates Analysis (PCoA) based on Bray–Curtis dissimilarities depicting fungal community composition across conventional (green), organic (blue) and wild (red) bog types in 2021 **(C)** and 2022 **(D)**. Each point represents a sample, and ellipses denote 95% confidence intervals for each bog type.

Alpha Diversity: In 2021, Shannon diversity differed significantly among the bog types, as indicated by the Kruskal–Wallis test (p< 0.05). Pairwise comparisons revealed a significant difference between conventional and organic bogs at the 5% level (p = 0.026), and a marginally significant difference between conventional and wild bogs at the 10% level (p = 0.087). In contrast, no significant differences in Shannon diversity were observed among the three bog types in 2022. Although wild samples exhibited slightly higher average diversity than conventional and organic samples, these differences were not statistically significant (**Figures 3A, B**).

Beta Diversity: Principal Coordinates Analysis (PCoA) based on Bray–Curtis dissimilarities revealed distinct clustering patterns of fungal communities by bog type. This pattern was confirmed by a permutational multivariate analysis of variance (PERMANOVA), which indicated significant difference in community composition among conventional, organic, and wild bogs and study years on community structure (R² = 0.25, p = 0.001). In 2021, PERMANOVA indicated a marginally significant difference (at 10% level of significance) in community composition among conventional, organic, and wild bogs (R² = 0.117, *F* = 1.93, *p* = 0.066), with PCoA1 and PCoA2 explaining 35.07% and 26.70% of the variation, respectively (**Figure 3C**). In contrast, fungal communities differed more significantly (at 5% level) among bog types in 2022 (R² = 0.224, *F* = 4.18, *p* = 0.001), with PCoA1 and PCoA2 accounting for 33.40% and 26.47% of the variation, respectively (**Figure 3D**).

### 3.4 Year-to-year variations in CFR fungi

**Figure 4:**
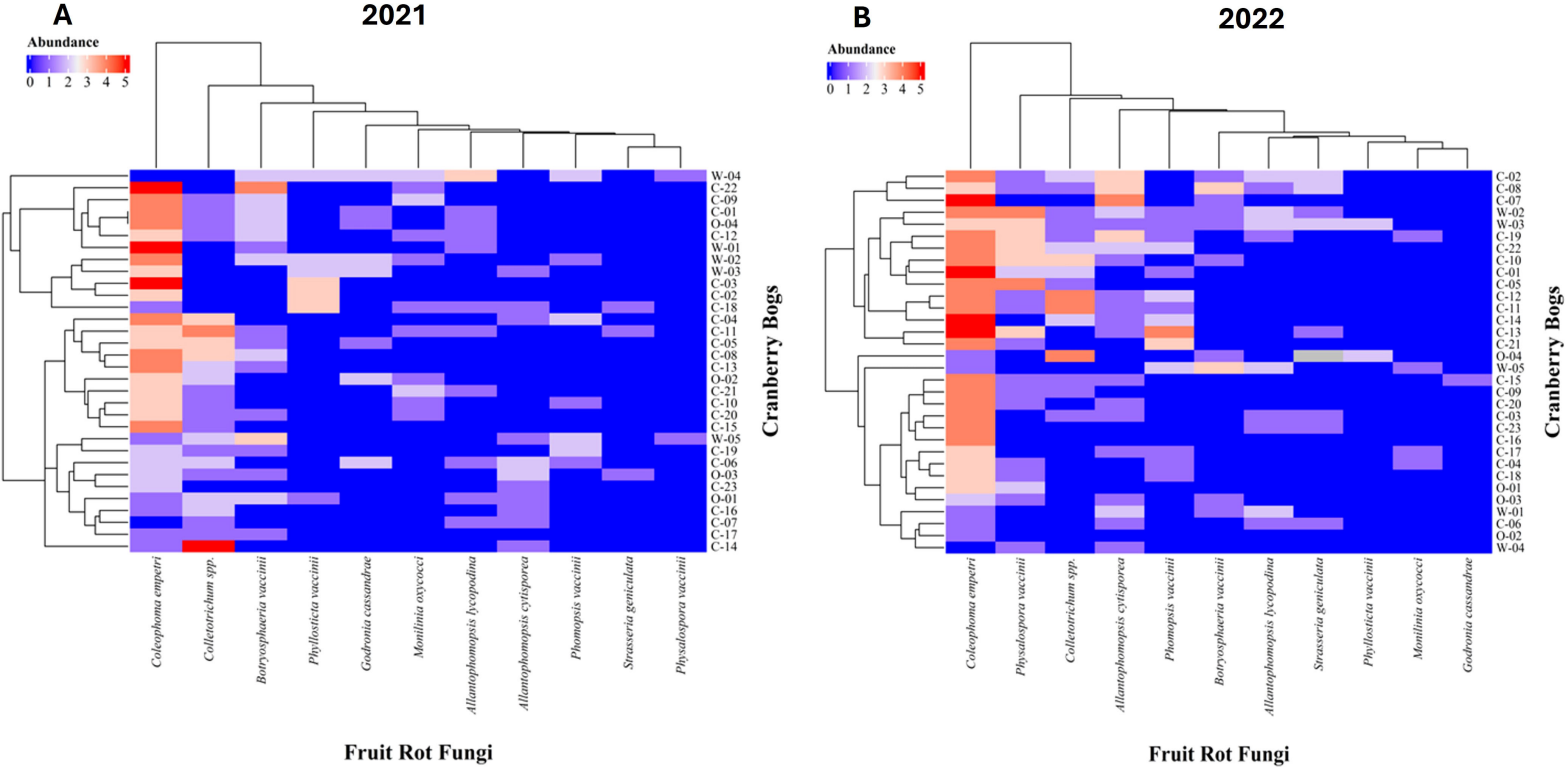
Heatmap showing the abundance of fungi associated with cranberry fruit rot across various cranberry systems sampled in **(A)** 2021 and **(B)** 2022. The x-axis represents different fruit rot fungi, while the y-axis represents individual cranberry bogs studied. Letters at the beginning of bog name represent the bog type; C-Conventional, O-Organic and W-Wild. The color gradient from blue to red indicates the relative abundance of each fungus, with blue representing lower abundance and red indicating higher abundance. Dendrograms on both axes illustrate hierarchical clustering, grouping similar cranberry bogs and fungi based on their abundance patterns.

The abundance of fungi associated with cranberry fruit rot varied significantly between the years of study in 2021 and 2022 among the bogs (**Figures 4A, B**). The heatmap reveals notable differences in the abundance of various fungal species across cranberry bogs.

The proportion of bogs with consistent presence of cranberry fruit rot (CFR) fungi from one year to the next was higher in wild bogs compared to organic or conventional bogs (**Table 5**). Wild bogs exhibited a greater rate of CFR fungal carryover, with 7 out of the 11 CFR fungi persisting from one year to the next (**Table 5**). The highest persistence was observed for *Coleophoma empetri*, with 60% of wild bogs showing carryover across both years. *Physalospora vaccinii*, *Phyllosticta vaccinii*, and *Allantophomopsis cytisporea* were detected in 20% of wild bogs. Furthermore, 40% of wild bogs demonstrated consistent incidence of *Allantophomopsis lycopodina*, *Phomopsis vaccinii*, and *Botryosphaeria vaccinii*.

**Table 5:** Proportion of Conventional, Organic, and Wild Bogs with Consistent Prevalence of Individual Cranberry Fruit Rot Fungi in 2021 and 2022.

Fungi	Conventional %	Organic %	Wild %
*Allantophomopsis cytisporea*	13	25	20
*Allantophomopsis lycopodina*	4	0	40
*Strasseria geniculata*	0	0	0
*Botryosphaeria vaccinii*	4	25	40
*Coleophoma empetri*	96	100	60
*Colletotrichum spp.*	43	0	0
*Godronia cassandrae*	0	0	0
*Monilinia oxycocci*	0	0	0
*Phomopsis vaccinii*	9	0	40
*Phyllosticta vaccinii*	0	0	20
*Physalospora vaccinii*	0	0	20

In organic bogs, 3 out of the 11 CFR fungi persisted from one year to the next (**Table 5**). 25% organic bogs showed persistent incidence of *A. cytisporea* and *B. vaccinii* in both years, while *C. empetri* was detected in all bogs in both years. In conventional bogs, 6 out of the 11 CFR fungi persisted across both years (**Table 5**). *C. empetri* and *Colletotrichum* spp. exhibited higher persistence, being detected in 96% and 43% of bogs, respectively, while the persistence of other CFR fungi from 2021 to 2022 remained below 20% (**Table 5**).

## 4 Discussion

Cranberry fruit rot (CFR) is a complex and persistent disease associated with a diverse assemblage of fungal taxa, and it remains a major challenge across all major cranberry-growing regions. Despite its economic significance, the diversity, spatial distribution, and prevalence of CFR-associated fungi across different ecosystems—conventional, organic, and wild—are not well understood. Gaining insight into these patterns is essential for developing targeted and effective disease management strategies. This study addressed that knowledge gap by investigating CFR fungal communities across cranberry ecosystems in Massachusetts over two growing seasons (2021 and 2022).

Despite Massachusetts being the second-largest cranberry producer, with 4,694 hectares (11,600 acres) under cultivation, organic cranberry farming remains limited, accounting for less than 40 hectares and operating on relatively small parcels (< 8 hectares) and young farms (<30 years old) (Farm Credit East, 2023; USDA-NASS, 2023; Woods Hole Group, 2003). The inclusion of only four organic bogs in both study years reflects the challenges of cultivating cranberries under organic practices due to high disease pressure (Sandler et al., 2010). In contrast, 23 conventional bogs in 2021 and 40 in 2022 were included. Additionally, four wild bogs, situated in natural environments with minimal human intervention, provided a unique perspective on the CFR disease complex compared to managed systems.

The current study focused on 11 fungi commonly associated with CFR. While Koch’s postulates have only been confirmed for a few (e.g., *Phyllosticta vaccinii*), most remain “presumed CFR pathogens” due to their frequent isolation from rotted fruit without formal pathogenicity verification. *Godronia cassandrae* was more recently confirmed as a pathogen (Oudemans et al., 1998; Conti et al., 2020). Interestingly, *Phom. vaccinii*, viscid rot fungus, which was also previously been associated with cranberry upright dieback disease (Caruso, 2000), was consistently absent in organic bogs but present in wild and conventional systems, suggesting potential sensitivity to organic management. All other fungi were detected across all systems. Previous studies from Massachusetts (e.g., Bergman and Wilcox, 1936; Caruso and Ramsdell, 1995; Shear et al., 1931) and from other growing regions (Olatinwo et al., 2003; Polashock et al., 2009, 2017; Wells-Hansen and McManus, 2017; Wood et al., 2023) have consistently implicated these fungi in CFR. Our findings build upon this work by confirming their prevalence across a broader range of ecosystems and by using a molecular multiplex PCR approach (Conti et al., 2019), which offers improved detection accuracy compared to traditional culturing.

Fungi associated with ripe rot (*C. empetri*), black rot (*A. cytisporea*), berry speckle (*B. vaccinii*), and blotch rot (*Phys. vaccinii*) were consistently detected across all bog types. Notably, *C. empetri*, *B. vaccinii*, and *A. cytisporea* showed persistence across years in all systems, supporting their status as dominant and ecologically well adapted CFR fungi. *Colletotrichum* spp. were consistently abundant in conventional systems but appeared in only one of the two years in wild and organic systems. While the CFR fungal patterns observed in conventional bogs align with findings from Oudemans et al. (1998) and Polashock et al. (2009), our results uniquely highlight the ability of these fungal species to persist in unmanaged or less intensively managed environments.

Significant differences were observed in the prevalence of cranberry fruit rot (CFR) fungi across cranberry ecosystems and between the two study years. Year-to-year variation and analysis of fungal carryover revealed that a higher proportion of wild bogs maintained CFR fungi from one year to the next—with 7 of the 11 fungi persisting—compared to 4 in organic and 6 in conventional bogs. Wild bogs also exhibited the highest species richness, averaging five fungal species annually, compared to four in both conventional and organic bogs. This may be attributed to the absence of interventions or cultural practices in wild bogs, in contrast to conventional and organic systems. Domestication is known to alter plant interactions with other organisms, thereby influencing agroecosystem dynamics (García-Palacios et al., 2013; Leff et al., 2016; Nguyen et al., 2015). The persistence of CFR fungi from one year to the next has similarly been documented in previous studies (Oudemans et al., 1998).

While species richness remained stable in conventional and wild bogs across both years, a marked decline was observed in organic bogs in 2022. Notably, *Physalospora vaccinii* (Phys. vaccinii) exhibited a dramatic increase in prevalence—from 0% in 2021 to 71.79% in 2022—representing the most substantial year-to-year shift among the CFR fungi studied. Furthermore, despite most growers using similar fungicide programs and frequencies, fruit rot incidence in conventional bogs ranged from 2–42% in 2021 and 1–48% in 2022. These findings highlight the dynamic nature of CFR fungi and the likely influence of temporal and management-related factors. Such variation may reflect differences in fungal species composition, inoculum pressure, environmental conditions, microclimate (e.g., canopy density, light penetration), and agronomic practices such as fungicide application timing, pruning, sanding, irrigation, and fertilizer use (Averill et al., 2008; Caruso and Ramsdell, 1995; Caruso et al., 2000; Oudemans et al., 1998). Unfortunately, detailed data on cultural practices were not available for the fields included in this study, limiting our ability to directly link specific practices to CFR dynamics. To fully assess the impact of domestication and fungicide use on CFR fungal communities, long-term, multi-region studies across wild, organic, and conventional systems are essential. These studies should focus on evaluating a broad range of ecological and agronomic factors that influence CFR incidence and fungal community composition. Understanding whether these influences are ultimately beneficial or detrimental will be critical for refining disease management practices and improving fruit quality.

Additionally, the lack of rot incidence data from wild bogs (due to natural heterogeneity) and organic bogs (due to limited pre-harvest access) represents a notable limitation, constraining our ability to correlate fungal profiles with disease severity in these systems. Nonetheless, the observed patterns of species richness and fungal persistence across ecosystems provide valuable ecological insight. To address this gap, future studies should prioritize developing standardized methods for evaluating rot incidence in organic and wild systems, potentially through direct field-level assessments or remote sensing technologies such as drone imagery. These approaches will enhance our understanding of CFR dynamics and improve our ability to predict disease risk across diverse cranberry production systems.

The observed differences in fungal diversity and community composition across bog types and years highlight the complex interplay between management practices, environmental variability, and microbial ecology in cranberry production. In 2021, Shannon diversity varied significantly among bog types, with conventional bogs exhibiting lower fungal diversity compared to organic and wild systems. These findings are consistent with broader research indicating that intensive management practices—including the frequent use of synthetic fungicides and fertilizers—can reduce microbial diversity by selectively suppressing sensitive taxa (Hartmann et al., 2015; Schlatter et al., 2017). In contrast, organic and wild systems, which generally operate with minimal or no chemical inputs, tend to support richer and more heterogeneous microbial communities (Liu et al., 2020). This may result from reduced disturbance, increased plant–microbe and microbe–microbe interactions, and higher soil organic matter content.

In 2022, however, no significant differences in alpha diversity were observed among bog types, although wild bogs again showed a trend toward higher diversity. This interannual variability may be explained by fluctuations in environmental conditions such as temperature, humidity, and precipitation, which are known to affect fungal development and dispersal (Chagnon et al., 2020; Karlsson et al., 2014). The inconsistency between years emphasizes the limitations of single-year studies and supports the need for multi-year monitoring to capture the temporal dynamics of fungal communities—particularly in perennial crops like cranberry, where seasonal variation in management intensity and plant phenology is common.

Beta diversity analyses further confirmed differences in fungal community composition among bog types, with stronger differentiation observed in 2022. These patterns suggest that both management practices and environmental conditions contributed to shaping community structure. PCoA plots revealed tighter clustering in conventional bogs and greater dispersion in wild bogs, indicating more homogeneous fungal communities in intensively managed systems and greater heterogeneity in unmanaged or less disturbed environments. This observation is consistent with previous work showing that agricultural intensification tends to homogenize soil and plant-associated microbiota (Zhou et al., 2020).

Importantly, wild bogs consistently harbored more diverse and distinct fungal communities than managed systems. These natural landscapes may function as reservoirs of microbial diversity, including potential biocontrol agents or rare taxa that enhance ecological resilience and disease suppression (Mendes et al., 2011; Trivedi et al., 2020). Understanding the ecological functions of these taxa could inform more sustainable and biologically integrated disease management strategies—particularly as cranberry growers contend with rising concerns about fungicide resistance and regulatory restrictions on chemical use.

The current study aimed at characterizing the 11 most prevalent CFR fungi (McManus et al., 2003; Oudemans et al., 1998) using multiplex PCR (Conti et al., 2019). In contrast, earlier studies conducted in Massachusetts (Bergman and Wilcox, 1936; Shear et al., 1931; Stevens, 1924; Rudolph and Franklin, 1920; Zuckerman, 1958) employed traditional methods such as plating, pure culture isolation, and microscopy to examine a broad range of fungi associated with decayed cranberry fruits. A key distinction of this study is its focus on CFR fungi from across wild, organic, and conventional (Stevens’ cultivar) production systems. In comparison, historical research was limited to conventional bogs primarily planted with ‘Early Black’ and ‘Howes’ cultivars. Another notable difference lies in the focus of disease assessment: while earlier studies examined both field rot (with symptoms appearing during fruit development and prior to harvest) and storage rot (rot developing post-harvest, during storage), our study focused exclusively on CFR fungi associated with field rot. This focus is more relevant to current industry practices, as over 90% of cranberries are now frozen upon arrival at the handlers’ (processing industry) Receiving Station and eventually processed into value-added products, thereby reducing the significance of storage rot in modern production systems. Despite methodological and temporal differences, historical studies from Massachusetts (Bergman and Wilcox, 1936; Shear et al., 1931; Stevens, 1924; Rudolph and Franklin, 1920; Zuckerman, 1958), consistently reported *Glomerella*, *Godronia*, *Guignardia*, *Sporonema*, *Diaporthe*, *Fusicoccum putrefaciens*, and *Pestalotia vaccinii* as dominant rot-associated fungi. More recently, Polashock et al. (2009) identified *Physalospora vaccinii*, *Colletotrichum acutatum, C. gloeosporioides, Phyllosticta vaccinii*, and *Coleophoma empetri* as the five most prevalent CFR fungi in the United States (including Massachusetts) based on results from samples collected from 18–24 fields each in 2005 and 2006 growing seasons.

Their (Polashock et al., 2009) study differs from the current work in that it examined the morphological and molecular variability of the five most prevalent CFR fungi (enlisted above) across four growing regions—Massachusetts, New Jersey, British Columbia, and Wisconsin—by analyzing the internal transcribed spacer (ITS) and large subunit (LSU) regions of ribosomal DNA. They reported isolating all five target fungi except *C. gloeosporioides* from Massachusetts, with only *C. acutatum* detected in that region. In contrast, our study focused on the 11 most frequently isolated CFR fungi using multiplex PCR. We did not distinguish between *C. acutatum* and *C. gloeosporioides*, limiting our ability to assess potential changes in their distribution since Polashock et al. (2009) survey. Nonetheless, both studies, along with earlier research studies, emphasizes the dynamic nature of CFR fungal complexes and their shifting prevalence in Massachusetts over the past century.

Similarly shifts in CFR fungal communities have been observed in other cranberry growing regions of the United States as well. Early surveys from New Jersey (1926–1929) identified *Phyllosticta vaccinii* as the primary cause of fruit decay (Oudemans et al., 1998). Later surveys (1994–1996) reported *Physalospora vaccinii* and *Glomerella cingulata* (syn. *Colletotrichum gloeosporioides*) as the most prevalent and widespread field-rotting fungi. Additionally, *Coleophoma empetri*, *Phyl. vaccinii*, and *Phom. vaccinii* were frequently isolated, although from limited locations (Stiles and Oudemans, 1999). A three-year survey (1999–2001) of eight commercial farms in Michigan by Olatinwo et al. (2003) found *C. acutatum*, *Pestalotia vaccinii*, and *Phyl. vaccinii* to be the most frequently recovered fungi from decayed fruit. Occasional isolations of *Fusicoccum putrefaciens* (syn. *Godronia cassandrae*), *Phom. vaccinii*, *Phys. vaccinii*, *A. lycopodina*, *C. empetri*, and *Botrytis cinerea* were also reported in 1999, with increased frequencies of *Phys. vaccinii*, *P. vaccinii*, and *C. empetri* in 2000.

With reference to cranberry growing regions in Canada, Conti et al. (2019) conducted a study using cranberry fruits randomly collected from four farms near Quebec City, Canada. Through a combination of pure culturing and multiplex PCR techniques, over 300 pure cultures were isolated and analyzed. Of these, only 23 were positively identified as one of the multiplex target fungi associated with CFR, with *Phys. Vaccinii*, *C. empetri*, *S. geniculata*, *Phyl. elongata*, *Phom. vaccinii*, and *C. fructivorum* being more abundant. In another large-scale study, Conti et al. (2022) investigated the presence of CFR fungi across different farming practices, sampling times, and cranberry cultivars, and found that four species—*G*. *cassandrae*, *C*. *fructivorum*, *A*. *cytisporea*, and *C*. *empetri*—consistently predominated across all variables. Wood et al. (2023) from British Columbia analyzed samples of flowers, green fruits, and ripe fruits from 28 farms across six regions in BC for CFR fungi using laboratory plating followed by ITS sequencing. Consistent with our findings, they reported significant variation in fungal isolations across farms and between years. All fungi we evaluated through multiplex PCR were frequently isolated except *A. lycopodina*, *S*. *geniculata*, and *Monilinia oxycocci*. Additionally, Wood et al. (2023) consistently isolated *B*. *cinerea*, *Alternaria*, *Aspergillus*, *Cladosporium*, *Epicoccum*, *Fusarium*, *Mucor*, *Penicillium*, *Pestalotiopsis*, and yeasts at low frequencies. Notably, *Mucor* and *Penicillium* spp. were also isolated by Conti et al. (2019). Collectively, all these studies suggest the dynamic nature of CFR fungal complexes, which has shifted over the past century which could be attributed to changes in cultivation practices, diagnostic methods, and environmental conditions.

The adoption of multiplex PCR represented a major leap forward in terms of efficiency and accuracy for CFR fungal detection (Conti et al., 2019, 2022). This method significantly reduced the time required for fungal detection compared to traditional approaches, which involve plating, isolating, microscopy, and uniplex PCR (Lievens and Thomma, 2007; Venbrux et al., 2023). By enabling the simultaneous detection of all CFR fungi in just a few reactions, the multiplex PCR streamlined the diagnostic process and improved detection precision, ensuring that the most relevant fungi were quickly identified. Additionally, its high sensitivity allowed for the detection of even low-abundance and near-obligate pathogens, such as *M. oxycocci* (Sanderson and Jeffers, 2001), which could have been missed using traditional plating methods which were successful in isolation of other major CFR fungi *in vitro* (Tadych et al., 2012; Polashock et al., 2009). However, despite these advantages, traditional methods still hold value, particularly in their broader scope of detection. The traditional approaches could have facilitated the isolation and identification of a wider range of fungi, not targeted by the current predefined multiplex panel. While the roles of these additional, uncharacterized fungi, are not yet fully understood, they may play a significant role in the CFR complex, either as emerging pathogens or contributors to disease dynamics. For example, Conti et al. (2019) reported the isolation of over 200 pure cultures from cranberries which had not previously been reported as pathogenic on cranberries before.

Future studies should aim to include complementary methods such as ITS-based metabarcoding that could capture a broader fungal spectrum and enhance understanding of disease ecology. Additionally, the multiplex PCR does not distinguish between closely related taxa, such as *Colletotrichum acutatum* and *C. gloeosporioides*, which may differ in pathogenicity or fungicide resistance. Future primer development should aim to resolve species-level distinctions within such complexes.

In this study, during initial method standardization some discrepancies were observed between mPCR and plating results, namely with higher detections for some CFR species (e.g. *Coleophoma empetri* for example) using mPCR. The mPCR is based on primers developed to amplify a part of ribosomal RNA gene, including ITS1 and ITS2. This genomic region, widely used for fungal identification, has admittedly limited species-level resolution in some genera due to its conserved nature (Baturo-Cieśniewska et al., 2020; Hyde et al., 2024). However, in the context of our study focusing on CFR fungi, we found ITS-based primers to provide a more reliable approach due to their stronger discriminatory capabilities across the diverse fungal species complexes typical of CFR. To mitigate the potential for false positives, rigorous efforts were made in the sampling protocol to minimize the detection of related non-target species. Specifically, only visibly rotten fruits were selected, and these were thoroughly washed and surface-sterilized prior to DNA extraction. This sampling strategy helps ensure that PCR diagnostics target only fungal species actively infecting cranberry fruit, thereby reducing the likelihood of contamination from closely related species. Additionally, the consistent isolation of these fungi from rotten cranberry fruit across years and bog types supports their ecological association with the CFR complex (Oudemans et al., 1998; Olatinwo et al., 2003; Polashock et al., 2009; Wood et al., 2023). On the other hand, plating techniques offer their own set of limitations, particularly when several potential fungi are present in a single sample. Variability in their abundance or differential growth capacity on culture media may result in false negatives. The necrotrophic and saprophytic fungi are particularly advantaged in these conditions, compared to biotrophic or hemi-biotrophic species (Akhtar et al., 2020). This being said, future research incorporating multilocus sequencing for finer taxonomic resolution could enhance the accuracy of mPCR-based diagnostics.

## 5 Conclusions

This two-year study provides a comprehensive assessment of cranberry fruit rot (CFR)-associated fungal communities across conventional, organic, and wild production systems in Massachusetts. Using multiplex PCR (mPCR), we detected and quantified key CFR fungi, demonstrating its value as a rapid diagnostic tool. However, mPCR is limited to a predefined species panel and cannot assess fungal viability or pathogenic potential. As many detected fungi remain “presumed pathogens,” culture-based methods and validation through Koch’s postulates are essential for confirming causal roles—particularly for dominant taxa such as *Coleophoma empetri*, *Allantophomopsis cytisporea*, *Botryosphaeria vaccinii*, and *Physalospora vaccinii*.

Despite a smaller number of sampled organic and wild bogs compared to conventional sites, these ecosystems provided valuable insights into fungal diversity and stability. Wild bogs harbored richer and more consistent communities year-to-year, while organic bogs showed a notable decline in richness in 2022. Colletotrichum spp. were inconsistently detected, mostly in conventional bogs, possibly reflecting differences in management intensity or environmental factors. Rot incidence data were only available for conventional bogs, limiting comparisons across systems.

The observed variability across systems and years highlights the influence of both management and environmental conditions on CFR community structure. These findings highlight the importance of long-term, ecosystem-specific monitoring. Future work should integrate mPCR with untargeted methods (e.g., ITS metabarcoding), environmental metadata, and standardized rot incidence assessments—especially in organic and wild systems. These findings emphasize the need for continued research into the ecological and environmental factors influencing CFR dynamics, along with the development of tailored management strategies to support growers in addressing fruit rot challenges sustainably.

## Data Availability

The raw data supporting the conclusions of this article will be made available by the authors, without undue reservation.

## Supplementary material

Supplementary Figure 1 Variability of fungal species detected in conventional, organic, and wild bogs. Box plots summarize the distribution of cranberry fruit rot fungal species isolated. Red dots represent the average number CFR fungal species causing rot in bog type.
