# Supplementary figures and images for: Comparative analysis of cranberry fruit rot fungal diversity in Massachusetts from wild, organic, and conventional ecosystems using multiplex PCR
Source: Front Plant Sci. 2025 Aug 15;16:1500877. doi: 10.3389/fpls.2025.1500877 (PMC12394219; doi:10.3389/fpls.2025.1500877)

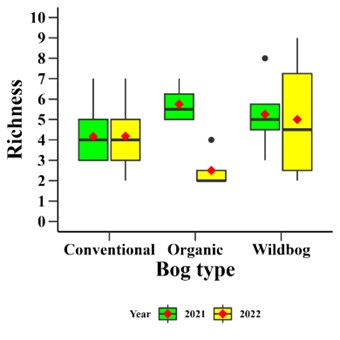

Supplement: Supplementary Figure 1 — Variability of fungal species detected in conventional, organic, and wild bogs. Box plots summarize the distribution of cranberry fruit rot fungal species isolated. Red dots represent the average number CFR fungal species causing rot in bog type. [file Image1.jpg]
